# Multimodality Imaging in HIV-Associated Cardiovascular Complications: A Comprehensive Review

**DOI:** 10.3390/ijerph20032201

**Published:** 2023-01-26

**Authors:** Parveen Kumar, Christophe Arendt, Simon Martin, Safaa Al Soufi, Philipp DeLeuw, Eike Nagel, Valentina O. Puntmann

**Affiliations:** 1 Institute of Experimental and Translational Cardiac Imaging, DZHK, Centre for Cardiovascular Imaging, University Hospital Frankfurt, 60590 Frankfurt am Main, Germany; 2 Department of Diagnostic and Interventional Radiology, University Hospital Frankfurt, 60590 Frankfurt am Main, Germany; 3 Infektiologikum, 60590 Frankfurt-am Main, Germany

**Keywords:** cardiovascular magnetic resonance, human immunodeficiency virus, myocarditis

## Abstract

Human immunodeficiency virus (HIV) infection is a leading cause of mortality and morbidity worldwide. The introduction of antiretroviral therapy (ART) has significantly reduced the risk of developing acquired immune deficiency syndrome and increased life expectancy, approaching that of the general population. However, people living with HIV have a substantially increased risk of cardiovascular diseases despite long-term viral suppression using ART. HIV-associated cardiovascular complications encompass a broad spectrum of diseases that involve the myocardium, pericardium, coronary arteries, valves, and systemic and pulmonary vasculature. Traditional risk stratification tools do not accurately predict cardiovascular risk in this population. Multimodality imaging plays an essential role in the evaluation of various HIV-related cardiovascular complications. Here, we emphasize the role of multimodality imaging in establishing the diagnosis and aetiopathogenesis of various cardiovascular manifestations related to chronic HIV disease. This review also provides a critical appraisal of contemporary data and illustrative cases.

## 1. Introduction

**Figure 1:**
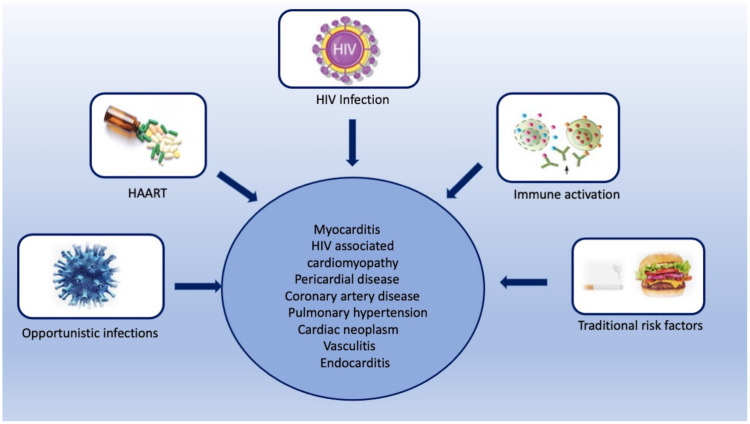
Central illustration showing various risk factors for various HIV-associated cardiovascular complications.

HIV infection represents a major public health concern. The introduction of potent antiretroviral therapy (ART) has distinctly reduced HIV-related mortality and morbidity [1]. However, with an increase in life expectancy, chronic complications related to HIV infection are becoming more evident. Cardiovascular disease is commonly found in HIV patients [2]. The high prevalence of cardiovascular risk factors in HIV-infected patients, together with ART-induced metabolic changes and an HIV-induced chronic inflammatory state, increases the risk of cardiovascular diseases [3,4]. The clinical expression of cardiovascular disorders is variable and depends on various factors, including the degree of immunodeficiency, associated opportunistic infections and drug interactions and toxicities. The presentation of myocardial disease ranges from subclinical inflammation on cardiovascular imaging to symptomatic heart failure (Figure 1) [5]. Cardiac imaging plays an integral role in the assessment of HIV-related cardiovascular complications. Transthoracic echocardiography (TTE) is the primary tool for assessing myocardial function. It is cost-effective, readily available, and robust in identifying both systolic and diastolic dysfunctions [6]. Coronary CT angiography (CTA) is primarily used for assessing both clinical and subclinical coronary artery disease (CAD) in HIV-infected patients [7]. Cardiovascular magnetic resonance (CMR) has emerged as an important imaging tool. It not only yields anatomic and functional cardiac information, but also helps in tissue characterization by assessing the degree of oedema and fibrosis [8]. The current review summarizes the pathogenesis of various cardiovascular manifestations of HIV and discusses the role of multimodality imaging in establishing their diagnosis. 

## 2. HIV-Associated Cardiovascular Complications

The cardiovascular complications of HIV include a broad range of cardiac, coronary, valvular, and vascular manifestations. The pathogenesis and imaging findings of these complications are summarized in the following sections.

### 2.1. Myocarditis

Myocarditis is seen in approximately one-third of HIV-infected patients in histopathology, without any specific cause in more than 80% of the cases [9]. The proposed etiologies for myocarditis include direct invasion by the HIV virus, opportunistic infections, and HIV-mediated indirect pathways of myocardial inflammation [10,11,12,13,14]. HIV also catalyzes a series of indirect pathways that induce myocardial inflammation and myocardial damage [15,16,17]. Myocardial damage resulting from these indirect inflammatory pathways leads to left ventricular systolic dysfunction and hemodynamic compromise [18].

**Figure 2:**
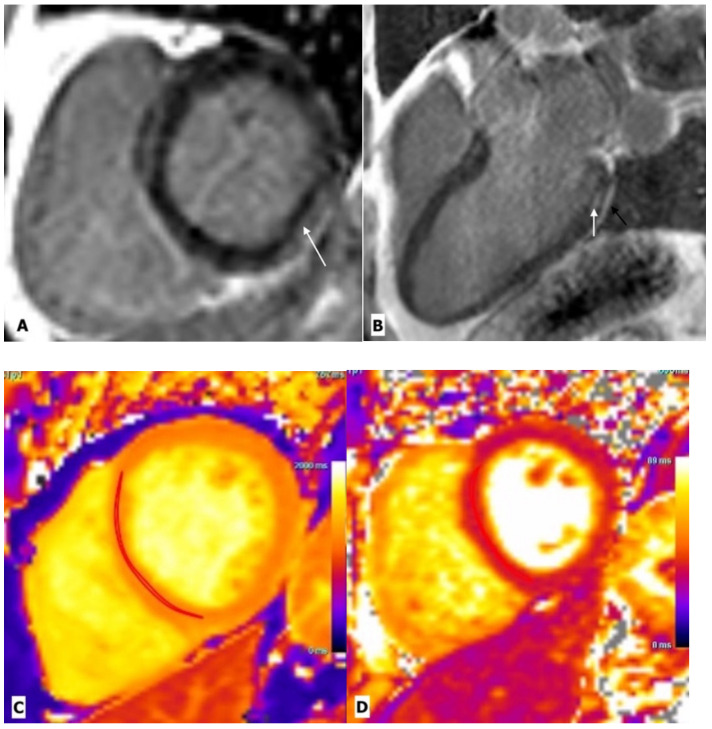
Cardiac magnetic resonance imaging in a 43-year-old man with HIV-related perimyocarditis. (**A**) Mid-ventricular short-axis and (**B**) 3-chamber long-axis images showing linear mid-wall enhancement in the basal inferior and inferolateral lateral walls (white arrows). The overlying pericardium also shows mild thickening and enhancement. (**C**) T1 map and (**D**) T2 map at the mid-LV show a markedly elevated myocardial T1 time of 1214 ms (normal 1104 ms at 3.0 Tesla) and T2 time of 44 ms (normal 37.4 ms at 3.0 Tesla) consistent with diffuse myocardial fibrosis.

TTE is a useful tool in the diagnostic assessment of suspected myocarditis. TTE investigates the wall thickness, chamber volume, and systolic and diastolic dysfunctions, and thus does not provide any direct evidence of myocarditis [19] (Table 1). CMR is a promising tool for assessing myocardial inflammation. Comprehensive CMR imaging not only yields anatomic and functional cardiac information, but also provides information on myocardial tissue composition, including the degree of oedema, inflammation, and fibrosis (Figure 2). Several cardiac imaging studies have provided deep insight into the structural and functional alterations associated with HIV-related myocarditis. CMR parameters indicating myocardial inflammation are elevated in HIV-infected patients, even with subclinical diseases [20,21]. Myocardial functional alteration (systolic or diastolic dysfunctions) may be a result of this higher inflammatory burden [20]. Julian et al. evaluated cardiovascular involvement in asymptomatic HIV-infected patients using CMR. When compared with healthy controls, HIV-infected patients showed a reduced left ventricular ejection fraction and lower global peak strain values. CMR parameters of myocardial inflammation such as the native T1 and relative T2 signal intensity ratios were elevated in HIV-infected patients. Late gadolinium enhancement suggesting focal myocardial fibrosis was seen in midventricular and basal inferolateral subepicardial regions in 27.3% of healthy controls [20]. Ntobeko et al. conducted a similar study using CMR to detect subclinical inflammation and myocardial disease in asymptomatic HIV-infected individuals. Compared to healthy controls, HIV-infected patients showed elevated myocardial mass, reduced left ventricular ejection fraction, a lower peak diastolic strain rate, and higher native T1 values. Furthermore, myocardial fibrosis and pericardial effusions were common in HIV-infected subjects [21]. Both these studies indicate that CMR markers of myocarditis are elevated even in subclinical inflammation; therefore, CMR may reveal a high burden of cardiovascular disease in asymptomatic HIV-infected patients.

The presence of diastolic dysfunction in asymptomatic patients has also been observed in various TTE-based studies [22,23]. A study by Hsue et al. demonstrated a high prevalence of diastolic dysfunction in asymptomatic HIV-infected individuals. After adjustment for age and various traditional risk factors, HIV-infected individuals had 2.4-times greater odds of having diastolic dysfunction compared to controls [22]. CMR is also helpful in monitoring treatment response in HIV-related myocarditis. In a prospective CMR-based study conducted by Robbertse et al., a contemporary HIV-infected cohort was evaluated before and 9 months after the initiation of HAART. The study reported a significant decrease in native T1 and extracellular volume (ECV) after 9 months of HAART, which was significantly associated with a decrease in C-reactive protein, a decrease in HIV viral load, and an improvement in CH4 count [24]. CMR has also emerged as a robust technique for prognostic stratification. Like other ischemic and non-ischemic cardiomyopathies, CMR has been evaluated for predicting adverse cardiovascular outcomes in HIV-infected individuals. A prospective observational study assessed the prognostic association between CMR parameters and cardiovascular outcomes in HIV-infected individuals on HAART. It was observed that patients with higher ECV values have a higher rate of cardiovascular outcomes [25]. In conclusion, while TTE is a primary screening modality, multiparametric CMR not only provides the opportunity to identify subclinical diseases in HIV-infected individuals, but also aids in treatment monitoring and prognostication. 

### 2.2. HIV Associated Cardiomyopathy

HIV infection is recognized as an important cause of dilated cardiomyopathy [26,27,28]. It is usually seen in the late stages of diseases and is associated with a low CD4 count [26]. The proposed etiological factors for HIV-associated cardiomyopathy include myocarditis, opportunistic infections, micronutrient deficiency, cardiac autoimmunity, and antiretroviral toxicity [29]. Patients with HIV-related cardiomyopathy show poor prognosis, with a reported median survival of 101 days, compared to a median survival of 472 days in patients with similar severity of HIV infection but normal hearts [30]. The gross pathological examination may show eccentric hypertrophy with dilated chambers and increased wall thickness. Conversely, there may be ventricular wall thinning. Other common features include endocardial fibroelastosis, apical mural thrombi, infective endocarditis, and apical mural thrombi [31]. 

TTE is the primary modality for assessing cardiomyopathy. In the pre-ART era, HIV-associated cardiomyopathy was recognized as systolic dysfunction with LV dilatation on TTE. However, after the onset of ART, HIV-associated cardiomyopathy is largely recognized as subclinical diastolic dysfunction [32]. Right ventricular (RV) dysfunction is common in HIV-infected patients, especially with dilated cardiomyopathy or HIV-associated pulmonary hypertension. Isolated RV dysfunction has been reported in both the pre-ART and ART eras [33]. A large contemporary study has shown that RV dysfunction in HIV-infected patients does not correlate with LV systolic dysfunction or elevated pulmonary artery pressure, suggesting that RV dysfunction may occur independently of LV dysfunction or pulmonary hypertension [33]. CMR provides reproducible measurements of cardiac function and tissue characterization in HIV cardiomyopathy [25]. A recent study conducted by DeLeuw et al. assessed the prognostic value of CMR in HIV-infected patients. The investigators used multiparametric CMR to assess myocardial volume, function, LV mass, perfusion, and myocardial scarring in 156 patients. These patients were subsequently followed for adverse cardiovascular events for a median follow-up of 13 months. It was found that patients with adverse events had higher native T1, native T2, and LV mass indexes. Furthermore, in multivariate analysis, native T1 was found to be an independent predictor of adverse events, whereas the conventional risk factors were not [25]. As there is a growing emphasis on identifying patients in pre-HF stages, the diagnostic and prognostic role of CMR may help in the personalized care of people living with HIV by screening for heart failure and allowing intervention with anti-remodeling treatment in early HF.

### 2.3. Pericardial Diseases

**Figure 3:**
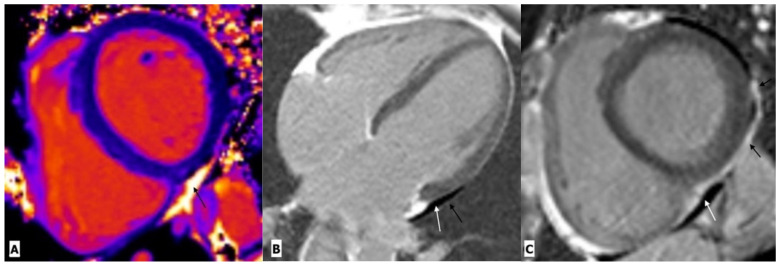
Cardiac magnetic resonance imaging in a 38-year-old man with HIV-related pericarditis and pericardial effusion. (**A**) Mid-ventricular short-axis view T1 map showing high signal intensity pericardial effusion (black arrow). (**B**) Four chambers view and (**C**) mid-ventricular short-axis view gadolinium enhancement images showing mild pericardial effusion (white arrow) with thickening and enhancement of pericardium (black arrow) along the left lateral LV wall.

The various HIV-related pericardial diseases include pericardial effusion, cardiac tamponade, pericarditis, and constrictive pericarditis [28,34,35,36,37,38,39,40,41]. Pericardial effusion was one of the common cardiac manifestations of HIV in the pre-HAART (highly active anti-retroviral therapy) era between 1985 and 1995, with a reported prevalence ranging from 5% to 45% in three major trials [28,34,35]. The etiology of pericardial effusion is unknown in asymptomatic patients, as they rarely need pericardiocentesis. Among symptomatic cases, infection and malignancy constitute approximately two-thirds of the cases. Other rare causes include chronic kidney diseases, hypothyroidism, and connective tissue diseases [36]. In developing countries, the most common cause of infective pericardial effusion is tuberculosis [37,38], while in developed countries, the common infective agents include streptococcus pneumoniae, staphylococcus aureus, chlamydia, listeria, and cryptococcus [39,40]. A common cause of malignant pericardial effusion is AIDS-related Kaposi sarcoma [41]. Serous pericardial effusion appears anechoic on echocardiography, hypodense on CT, and shows low T1 and high T2 signal intensity in MR images [42]. The purulent or sanguineous effusion shows floating echoes on echocardiography and appears iso-hyperdense on CT, with a high signal on T1-weighted MR images. Pericarditis is characterized by the thickening and enhancement of the pericardium (Figure 3) [42]. In routine clinical practice, TTE is the primary imaging modality for pericardial-related pathologies, while CT and CMR should be reserved for patients with difficult echo windows and constrictive pericarditis.

### 2.4. Coronary Artery Diseases

HIV-infected patients are at higher risk of coronary artery disease (CAD), with an estimated 1.5- to-2-fold higher risk compared to individuals without HIV [3,43]. The exact pathophysiology of the increased risk of CAD is not well understood. Previously, a higher prevalence of traditional risk factors such as smoking, substance abuse, and lipid abnormalities drew attention in HIV-infected patients [44,45]. Initial studies also focused on the positive association between lipid dystrophies and some ART medications [46]. However, recent studies show that HIV-positive patients are at significantly higher risk of non-calcific plaque compared to non-infected age-matched controls, even after adjustment for traditional risk factors [7]. Likewise, there is growing evidence that HIV-infected patients, whether on or off HART, develop subclinical atherosclerosis [47]. These findings have shifted the paradigm on the role of traditional risk factors and HART in the pathogenesis of CVD. It has also led to the consensus that chronic inflammation and immune dysfunction are major factors that accelerate atherosclerosis and plaque rupture in HIV [48].

Non-invasive coronary imaging allows early detection of atherosclerosis in patients with HIV by utilizing advances in both CT and MRI. The coronary artery calcium (CAC) score is a strong predictor of CAD and provides incremental risk stratification for major adverse cardiovascular events over traditional risk factors in the general population [49]. The utility of CAC has also been explored in HIV-infected individuals. In a prospective observational study of 843 HIV-infected patients with a median follow-up of 2.8 years, it was seen that a CAC score of 100 was associated with 3.3-times higher odds of myocardial infarction, independent of gender and age [50]. A potential limitation of CAC screening is its inability to identify non-calcific plaque [51]. CCTA is an established tool for characterizing coronary artery plaque. Plaque with low attenuation, spotty calcification, and positive remodeling is vulnerable and is associated with an increased risk of acute coronary syndromes [52]. A study showed an increased prevalence of low-attenuation coronary plaque in HIV-positive men compared to age-matched HIV-negative healthy controls. The increased prevalence of low-attenuation plaque could explain the higher rates of acute coronary syndrome in HIV-infected patients [53]. Hoffman et al. assessed the prevalence and extent of CAD in HIV-infected patients using CTA. The results showed a high prevalence of noncalcified, nonobstructive, and vulnerable plaque in the HIV-infected cohort. The participants with plaque demonstrated higher levels of inflammation and immune-mediated markers, independent of traditional risk factors and HIV parameters. A substantial prevalence of CAD was seen in young patients with low traditional cardiovascular risk factors [54]. This study highlights the key role of arterial inflammation and immune activation in HIV-related CAD, independent of traditional risk factors. Further studies are needed to determine the effects of statin therapy on modulating these pathways and reducing plaque in this population. Although CTA is a non-invasive method of investigating early atherosclerosis in the adult population, its use is limited in children because of concerns about radiation exposure. 

**Figure 4:**
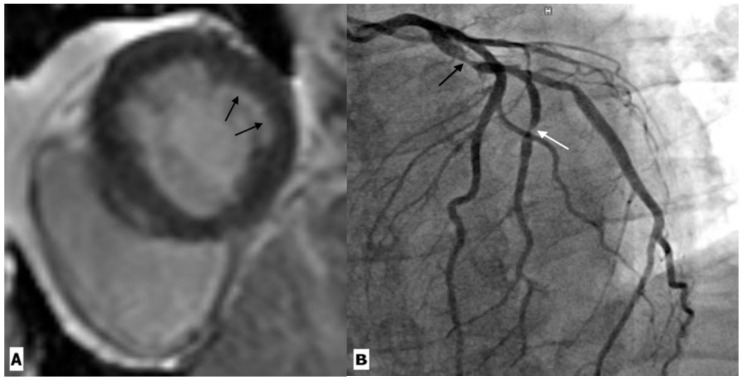
Cardiac magnetic resonance imaging in a 59-year-old HIV-infected male patient with coronary artery disease. (**A**) Basal ventricular short-axis view gadolinium enhancement image showing subendocardial enhancement in the inferolateral segment (black arrow). (**B**) Coronary angiography revealed severe stenosis in the proximal left circumflex artery (black arrow) and its first obtuse marginal branch (white arrow).

**Figure 5:**
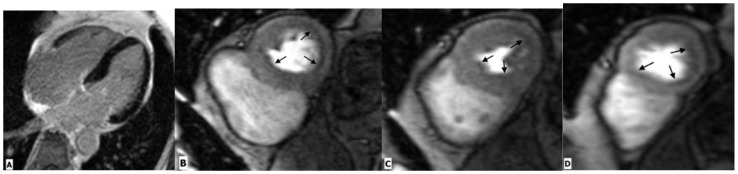

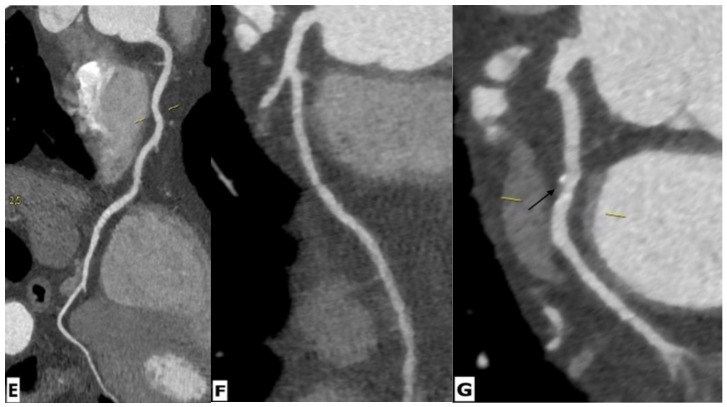
Cardiac magnetic resonance imaging in a 65-year-old HIV-infected male patient with microvascular coronary dysfunction. (**A**) Mid-ventricular short-axis late gadolinium enhancement image showing no ischemic pattern. Stress CMR was performed via regadenoson administration. (**B**–**D**) Stress protocols images of three ventricular slices (basal, mid-ventricular, and apical slices) show diffuse subendocardial hypoperfusion (arrows) due to microvascular dysfunction. (**E**–**G**) Multiplanar reformatted contrast-enhanced computed tomography images showing the right coronary artery (RCA), left anterior descending artery (LAD), and left circumflex artery (LCx), respectively. RCA and LAD are normal without any atherosclerotic diseases. LCx shows focal mixed plaque causing minimal luminal stenosis (arrow).

The introduction of 3T MR scanners provides an excellent opportunity to study early cardiovascular changes in the younger population with HIV infection. Irene J. et al. assessed the prevalence of CAD using MR angiography in HIV-infected children. The mean age of the study cohort was 18.9 ± 3.4 years with a range of 13–29 years. More than 50% of patients (14/27) showed evidence of CAD, suggesting early atherosclerosis [55]. Although it is difficult to make conclusions about specific risk factors given the small study sample, these preliminary data do highlight the importance of imaging in examining subclinical CAD. CMR imaging incorporates both morphological and functional characterization in ischemic heart disease by evaluating the myocardial function, myocardial oedema, ischemia, and scarring (Figure 4). Imaging of necrotic myocardium (acute infarction) and scarring (chronic infarction) is a hallmark of clinical CMR, the accuracy of which has been validated against histopathology in multiple animal studies. Stress myocardial perfusion CMR is helpful in detecting stress-inducible ischemia in suspected ischemic heart disease [56]. The latest studies have demonstrated that chronic infection, endothelial activation, and increased cardiovascular risk factors lead to the development of coronary microvascular dysfunction (CMD). CMD is a risk marker and can be used to study the effects of particular interventions on coronary microcirculation. In addition, the identification of microvascular disease helps characterize chest pain in patients with no epicardial coronary disease. In recent years, CMR has also emerged as a modality for microcirculation assessment [57]. Briefly, visual assessment is based on capturing the first-pass transit of gadolinium in the myocardium. A wide hypointense subendocardium in the absence of any scar or obstructive CAD is consistent with microvascular dysfunction [58] (Figure 5). Myocardial blood flow and myocardial perfusion reserve can also be calculated by rest and stress perfusion CMR [58]. In a nutshell, the diagnosis of CAD by CT or CMR is critical in understanding disease progression, which aids in early therapeutic interventions. Therefore, CTA should be used to identify CAD in HIV-infected patients with high traditional cardiovascular risk factors.

### 2.5. Pulmonary Hypertension

HIV is an established risk factor for pulmonary hypertension (PH), with a significantly higher prevalence of PH in HIV-infected individuals compared with the general population (0.5 vs. 0.0015%) [59]. It has been hypothesized that HIV-protein-related factors cause persistent activation of metabolic and proliferation signaling pathways, leading to pulmonary vascular remodeling. The resultant medial hypertrophy, perivascular cuffing, and thrombosis lead to PH. Other factors contributing to PH include illicit drug abuse and concomitant secondary pulmonary infections [60]. The most commonly observed symptoms include progressive shortness of breath, pedal oedema, and cough.

The imaging features of HIV-related PH are similar to primary hypertension. The common echocardiographic findings of PH include dilation of the right heart chambers (98%), tricuspid regurgitation (64%), and paradoxical septal motion (40%). Other echocardiographic findings include RV hypertrophy, abnormal septal motion, and pulmonary valve insufficiency [61,62]. CT angiography plays a crucial role in the diagnostic workup of pulmonary hypertension. The characteristic features of PH on CTA include central pulmonary artery dilatation, an abrupt decrease in the caliber of segmental and subsegmental arteries, and small tortuous peripheral vessels representing plexogenic arteriopathy. Additional CT features include right heart enlargement and a mosaic attenuation pattern of lung parenchyma [63,64]. CMR allows additional characterization of PH, providing information on RV morphology, chamber size, and RV systolic function. Abnormalities that can be seen with PH include RV hypertrophy, RV wall motion abnormalities, reduced RV ejection fraction, tricuspid regurgitation, and right atrial enlargement [65]. 

The diagnostic workup of PH also includes right heart catheterization (RHC), which is the gold standard for diagnosing PH. The European Society of Cardiology guideline recommends the assessment of patients with signs of RV dysfunction using TTE as a screening tool, with a confirmed diagnosis dependent on results obtained from RHC. The hemodynamic parameters measured by RHC include cardiac output, mean pulmonary artery pressure, pulmonary artery wedge pressure, right atrial pressure, right ventricular pressure, pulmonary vascular resistance, and cardiac index [66]. In addition to the diagnosis, these parameters also provide information on the disease severity, therapeutic response, and patient prognosis [67]. Each imaging technique provides incremental information with varying degrees of sensitivity and specificity. While chest X-ray and TTE are primary screening modalities, CT and CMR are used for lung parenchymal and biventricular dynamic assessment, respectively.

### 2.6. Cardiac Neoplasm

HIV-infected patients show an increased frequency of various malignancies. Commonly prevalent malignancies associated with HIV include Kaposi sarcoma (KS), non-Hodgkin lymphoma (NHL), Hodgkin disease, squamous cell carcinoma, and leiomyosarcoma [68,69]. Common cardiac malignancies include KS and NHL. 

KS was the first cardiac tumor seen in HIV-infected patients. It was first described in 1983 by Autran et al. in a young Haitian woman with AIDS [70]. The retrospective autopsy findings show a varying incidence of KS ranging from 12% to 28% [71,72]. In contrast to the classic KS seen in elderly Mediterranean men and endemic sub-Saharan African populations, HIV-associated Kaposi sarcoma is characterized by widespread dissemination and an aggressive nature [69]. HIV-associated KS usually involves the serous pericardium or subepicardial fat. There is a predilection for the subepicardial fat adjacent to the major coronary arteries, with or without the involvement of the adventitial layer of the pulmonary artery and ascending aorta [67]. Infiltration of the myocardium and coronary artery wall may occur [31]. Cardiac tamponade is a reported fatal complication of KS [73,74,75]. 

Cardiac lymphoma is the second most common tumor involving the heart in HIV patients, with an estimated relative risk of 72.8 [68,69]. In the majority of cases, such lesions are high-grade B-cell tumors, including large-cell immunoblastic lymphoma or small-cell Burkitt or Burkitt-like lymphoma. The right atrium is most commonly involved, followed by the right ventricle, left ventricle, left atrium, and interatrial and interventricular septae [31]. The pericardial extension is commonly seen. Patients present with pericardial effusion, cardiac tamponade, congestive heart failure, and cardiac arrhythmias [76,77,78]. On chest radiographs, cardiac lymphoma shows cardiomegaly, pericardial effusion, and signs of heart failure [79]. Echocardiography shows a hypoechoic, polypoidal cardiac mass with or without pericardial effusion [79]. On CT, cardiac lymphoma appears isodense to the myocardium and shows heterogenous contrast enhancement [80]. On CMR, cardiac lymphoma appears iso-hypointense on T1 and iso-hyperintense on T2, and depicts heterogeneous contrast enhancement. Central necrosis is not seen, unlike in other tumors such as angiosarcoma [80]. Pericardial effusion may be seen. There is scarce literature on the imaging features of HIV-related cardiac lymphoma, limited to case reports and case series only. In the case reported by Goldfarb et al., cardiac lymphoma was seen extensively filtrating and compressing the atria and main pulmonary artery, extending into the atrioventricular grooves and ventricular walls. There was contiguous involvement of the adjacent subcarinal mediastinum and superior vena cava as well [78]. In another case report, cardiac lymphoma was seen as a large, well-demarcated mass in the right atrium extending into the superior vena cava. The mass showed poor enhancement on CT and appeared iso-intense to the myocardium on MRI [81]. Another case reported by Llitjos showed infiltration of the right ventricular free and inferior wall and the interventricular septum. The lesion was seen encasing the entire right coronary artery [82]. The case reports highlight the importance of MRI as a useful tool to understand the characteristics and potential complications of the neoplasm, as it can explain conduction disorders by showing the septal extension of the infiltrative lymphoma. Imaging plays a central role in the diagnosis of cardiac tumors. Both CT and CMR provide essential information before any surgery. CT is useful in providing anatomical information, and CMR is advantageous for the tissue characterization of cardiac masses.

### 2.7. Vasculitis

HIV-infected patients show a range of inflammatory vascular conditions caused by both infective and non-infective etiologies. There is scarce literature on infective vasculitis. Large vessels such as the aorta can be involved, producing mycotic aneurysms, especially in intravenous drug abusers. The common causative agents include staphylococcus aureus, salmonella, and mycobacterium tuberculosis [83,84,85]. The non-infective type can involve both large and small vessels. Large vessel disease may be aneurysmal or occlusive. The aneurysmal type is more common and may involve multiple arteries such as the aorta and common iliac, femoral, popliteal, and common carotid arteries [86]. The occlusive type is less common and has been reported in a few case reports [86,87]. Small vessel vasculitis includes Henoch–Schönlein purpura, polyarteritis nodosa, and drug-induced hypersensitivity vasculitis. A few case reports have also described features similar to Kawasaki syndrome and Takayasu arteritis [88,89]. Ultrasound doppler is a non-invasive and cost-effective modality for evaluating large peripheral vessels. The common doppler findings include aneurysmal transformation of the carotid, aortic, femoral, and popliteal vessels [90]. CT and MR angiography is the preferred modality for small vessel vasculitis, which commonly involves the intracranial vessels [91]. Nuclear medicine-based techniques are another essential imaging tool for the early detection of vascular disease in HIV-infected patients [92]. One study has reported higher uptake of fluorodeoxyglucose on positron emission tomography/computed tomography in the aorta and carotid arteries of HIV-infected patients [93].

The selection of an appropriate imaging modality depends on the patient’s specific clinical presentation. While ultrasound doppler is primarily a screening tool for large peripheral vessel vasculitis, CT and MR angiography should be used for further characterizing the aneurysm and diagnosing small vessel vasculitis. 

### 2.8. Endocarditis

HIV-infected patients may develop infective or non-infective endocarditis. Infective endocarditis is usually seen in intravenous drug abusers, with a reported prevalence varying from 6.3% to 34% [26,94]. The clinical manifestations and survival rate of infective endocarditis in HIV-infected patients are similar to those in patients without HIV; however, late-stage HIV disease and infective endocarditis have a 30% higher mortality rate than asymptomatic HIV-positive patients [10,95]. The most common causative agent is staphylococcus aureus, followed by streptococcus viridians [95]. The clinical symptoms include fever, sweating, weight loss, and septic emboli. Possible complications include embolism; the left-sided endocarditis (mitral valve and aortic valve endocarditis) shows cerebral and myocardium embolism, while the right-sided endocarditis (tricuspid and pulmonary) shows pulmonary embolism. Another deleterious complication is the perforation of valvular leaflets or rupture of chordae tendinea, leading to acute valvular insufficiency and heart failure [96]. 

Valencia et al. undertook a retrospective analysis of 42 cases of infective endocarditis in HIV patients on HAART. The vegetation was detected by TTE, and the tricuspid valve was the most commonly affected valve (83%), followed by the mitral valve (9.7%), aortic valves (2.4%), and pulmonary valves (7.3%) [97]. There are case reports on non-valvular infective endocarditis in HIV infection. Bosch et al. reported the first case of septal infective endocarditis in an HIV patient caused by an atypical bacteria such as L. monocytogenes. TTE showed vegetation in the medial and apical portions of the septum with normal mitral or aortic valves [98].

The transesophageal echocardiogram (TEE) is another valuable tool for assessing endocarditis. Compared to TTE, TEE has shown higher diagnostic accuracy in detecting small vegetations of endocarditis [99]. A recent study has reported that TTE performs better than CT for detecting valvular infective endocarditis-related lesions (vegetations, erosion) and is similar to CT for detecting paravalvular IE-related lesions (abscess, pseudoaneurysm). Furthermore, the relatively lower cost of TEE compared to CMR and the absence of radiation exposure compared to CT also contribute to its wide-scale use in clinical practice [100]. The CT imaging findings of septic pulmonary embolism include bilateral lung nodular lesions ranging from 0.5 to 3.5cm, often with a basal predominance [101,102]. These nodules may be well-delineated or poorly defined and may show cavitation in up to 50% of cases. A feeding vessel sign is commonly seen if these nodules are located at the ends of pulmonary artery branches. There could be focal subpleural areas of consolidation showing peripheral rim-like enhancement. Additional findings include pleural effusion and mediastinal lymphadenopathy [101,102]. Non-bacterial thrombotic or marantic endocarditis is seen in HIV patients with end-stage diseases or with malignancy [103]. 

Multimodality imaging plays a pivotal role in the diagnosis of infective endocarditis. Both TTE and TEE can delineate the vegetation and have the added benefit of assessing the hemodynamic effects of valvular infection. Complementary CT, CMR, and nuclear imaging is occasionally required when there remains diagnostic uncertainty following echocardiography.

## 3. Conclusions

Cardiovascular complications are commonly seen in HIV-infected patients. The spectrum of these complications is broad and includes myocarditis, dilated cardiomyopathy, pericardial effusion, coronary artery disease, endocarditis, vasculitis, and cardiac tumors. Imaging plays an important role in the early diagnosis of these diseases. The selection of imaging modalities should be tailored to the clinical presentation in order to investigate the underlying cause and facilitate evidence-based treatment. 

## Figures and Tables

**Table 1:** Table summarizing the various cardiovascular manifestations of HIV.

Cardiovascular Manifestation	Authors	Type of Study	Number of Patients	Imaging Modality	Imaging Findings
Myocarditis	Luetkens, J.A. et al.	Prospective cohort study	28 patients and 22 control subjects	CMR	Compared with healthy controls, HIV-infected patients showed lower ejection fraction, lower global strain values, elevated native T1 and T2 values, and myocardial fibrosis, predominantly at the subepicardial of the midventricular and basal inferolateral wall.
	Ntusi, N. et al.	Cross sectional observational study	103 patients and 92 control subjects	CMR	Compared with controls, HIV-infected patients had lower LVEF, higher myocardial mass, lower peak diastolic strain rate, and higher native T1 values. Pericardial effusions and myocardial fibrosis were 3 and 4 times more common, respectively, in subjects with HIV infection.
	Robbertse et al.	Prospective study	73 patients and 22 healthy controls	CMR	Compared with controls, a significant decrease in native T1 and ECV was seen after 9 months of HAART in HIV patients, which was significantly associated with a decrease in C-reactive protein, a decrease in HIV viral load, and an improvement in CH4 count.
	De Leuw et al.	Prospective observational study	156 patients	CMR	Patients with higher ECV values have a higher rate of cardiovascular outcomes.
Ca rdiomyopathy	Sliwa, K. et al.	Prospective, registry study	518 HIV infected patients	CMR	The most common primary diagnosis attributable to HIV/AIDS was HIV-related cardiomyopathy (38%), followed by pericarditis/pericardial effusion (25%), HIV-related pulmonary hypertension (8.1%), and coronary artery diseases (2.4%).
	Simon et al.	Prospective multicentre study	104 HIV infected patients	TTE	Pulmonary hypertension (PAP>35 mm Hg) was seen in 15% patients, but RV dysfunction (RV fractional area change FAC < 35%) occurred in 11%. The study highlighted that RV dysfunction in HIV-infected individuals may be a separate entity from LV/global cardiomyopathy or pulmonary hypertension.
	de Leuw, P. et al.	Prospective observational longitudinal study	156 HIV infected patients	CMR	Patients with higher native T1, native T2, and LV mass indexes have higher events. In multivariable analyses, native T1 was independently predictive of adverse events. Traditional cardiovascular risk scores were not predictive of adverse events.
Pericardial diseases	Himelman, R.B. et al.	Prospective observational study	70 HIV infected patients	TTE	Pericardial effusion seen in 10% patients.
	Akhras, F. et al.	Prospective observational study	124 patients (101 with AIDS and 23 without opportunistic infection)	TTE	Pericardial effusion was more common in patients with AIDS (44%) as compared to HIV infected patients without opportunistic infections (9%).
Coronary artery disease	Raggi, P. et al.	Prospective observational study	843 patients	CT	In a median follow-up of 2.8 years, it was seen that a CAC score of 100 was associated with 3.3-times higher odds of myocardial infarction, independent of gender and age.
	Zanni, M.V. et al.	Cross sectional study	101 patients and 41 controls	CTA	The study showed an increased prevalence of low-attenuation coronary plaque in HIV-positive men compared to age-matched HIV-negative healthy controls.
	Hoffmann, U. et al.	Cohort study	755 HIV infected patients	CTA	Atherosclerotic plaque was seen in 49% patients. Luminal obstruction of at least 50% was rare (3%), but vulnerable plaque were more frequently observed (23%). Overall, 35% of patients demonstrated coronary artery calcium score scores greater than 0. IL-6. LpPLA2, oxLDL, and MCP-1 levels were higher in those with plaque compared to those without.
	Irene, J. et al.	Cross sectional study	27 HIV infected patients	MR angiography	More than half of the subjects showed CAD with luminal narrowing detected on MR angiography. There was no association between CAD and previous cardiac conditions (viral pericarditis and zidovudine related cardiomyopathy).
Pulmonary hypertension	Mehta et al.	Analytic review	131 HIV infected patients	TTE	The most common imaging finding was right heart chamber enlargement (98%), followed by tricuspid regurgitation (64%), and paradoxical septal motion (40%).
Cardiac Lymphoma	Goldfarb et al.	Case report	1	TTE, CT and CMR	CMR showed extensively filtrating mass lesion compressing the atria and main pulmonary artery and extending into the atrioventricular grooves and ventricular walls. There was contiguous involvement of the adjacent subcarinal mediastinum and superior vena cava as well.
	Shinro et al.	Case report	1	TTE, CT and CMR	CMR showed large well-demarcated mass in the right atrium extending to the superior vena cava. The mass showed poor enhancement with an iso-intense appearance to the myocardium on MRI.
	Llitjos et al.	Case report	1	CMR	CMR showed an infiltrating mass involving the right ventricular free and inferior walls and the interventricular septum. The lesion was seen encasing the entire right coronary artery.
Vasculitis	Gouny et al.	Case series	3	CTA	First case: a saccular aneurysm of the aorta whose neck was situated 3 cm proximal to the renal arteries and associated with a left lateral aortic hematoma; Second case: a fusiform aneurysm with a retroaortic extravasation suggesting chronic rupture and thickening of the anterior aspect of the aneurysmal wall; Third case: a fusiform aneurysm of the aorta extending to the common iliac arteries with its neck located 3 cm distal from the renal arteries.
	Sellami et al.	Case report	1	CTA	Early CT finding consisted of a slight-enhancing periaortic soft-tissue, while the aorta remained of normal size. Within two weeks, infection progressed to an infected aneurysm.
Endocarditis	Valencia et al.	Retrospective study	42	TTE	The tricuspid valve was the most commonly affected valve (83%), followed by the mitral valve (9.7%), the aortic valve (2.4%), and the pulmonary valve (7.3%).
	Bosch et al.	1	1	TTTE	TTE showed vegetation in the medial and apical portions of the septum with normal mitral or aortic valves.

## Data Availability

Data sharing not applicable, No new data were created or analyzed in this study. Data sharing is not applicable to this article.
